# Cross-lap suturing technique: a new strategy for repair of chronic perineal lacerations

**DOI:** 10.3389/fsurg.2026.1927783

**Published:** 2026-09-08

**Authors:** Liuxi Yang, Zhen Zhang, Weijun Peng, Fajuan Zheng

**Affiliations:** 1 College of Traditional Chinese Medicine, Chongqing Medical University, Chongqing, China; 2 Department of Anorectal Surgery, Chongqing Hospital of Traditional Chinese Medicine, Chongqing, China; 3 Science and Technology & Foreign Affairs Division, Chongqing Hospital of Traditional Chinese Medicine, Chongqing, China

**Keywords:** anal sphincter reconstruction, chronic obstetric anal sphincter injury, cross-lap suturing technique, end-to-end repair, fecal incontinence, overlapping sphincteroplasty

## Abstract

**Introduction:**

Chronic obstetric anal sphincter injuries (OASIS), particularly grade IIIb and IV perineal lacerations, often cause persistent fecal incontinence and severely impaired quality of life. Conventional overlapping sphincteroplasty is limited for chronic injuries due to muscle atrophy, fibrosis, high local tension and insufficient soft tissue coverage. This study presents a modified cross-suturing technique (Cross-lap) to optimize sphincter reconstruction and lower adverse tension distribution.

**Methods:**

Developed based on anatomical reconstruction principles, the Cross-lap technique is indicated for chronic or neglected OASIS. Its core step cross-folds external anal sphincter stumps and fixes them contralaterally in the dissected rectovaginal septal space. We describe its key surgical maneuvers, technical key points and preliminary clinical outcomes via a single illustrative case: a 58-year-old postmenopausal woman with chronic grade IV perineal laceration.

**Results:**

The Cross-lap suturing technique reconstructs the complete anal sphincter complex while decreasing bulky overlapping muscle tissue. Short-term follow-up of this illustrative case showed relieved fecal leakage, perianal dampness, anal fullness and impaired daily activity, alongside improved defecatory control. No severe early postoperative complications occurred, confirming its technical feasibility. Future large-cohort studies will adopt the validated Wexner score to objectively quantify functional recovery.

**Discussion:**

As a refined alternative to standard sphincteroplasty, this method redirects sphincter contractile force cephalad and mitigates stress concentration at the repair site. Such biomechanical benefits make it suitable for chronic OASIS patients with scarred, atrophic or fragile perineal tissues. Larger cohorts and extended follow-up are needed to verify its long-term clinical efficacy.

## 1 Introduction

Perineal laceration is a tear of the tissues between the vaginal opening and anus, most commonly occurring during vaginal delivery. Based on anatomical severity, these injuries are classified into four degrees, with third- and fourth-degree lacerations involving the anal sphincter complex collectively termed obstetric anal sphincter injuries (OASIS). Although most OASIS are repaired primarily after delivery, some patients develop chronic or neglected perineal lacerations due to delayed diagnosis, inadequate repair, or wound complications, leading to sphincter dysfunction, fecal incontinence, and impaired quality of life.

Surgical reconstruction remains the main treatment for symptomatic chronic OASIS. However, conventional overlapping sphincteroplasty may be challenging in chronic cases due to fibrosis, muscle atrophy, poor tissue quality, and increased repair-site tension, which may compromise healing. To address these limitations, we developed a modified cross-suturing approach termed the Cross-lap suturing technique, which integrates overlapping reinforcement and cross-fixation of external anal sphincter stumps to optimize force distribution and minimize tissue stress concentration.

This study describes the surgical principles and technical procedure of the Cross-lap suturing technique and reports its preliminary clinical application in chronic perineal laceration, providing a potential alternative approach for complex OASIS reconstruction.

## 2 Overview of perineal lacerations

Perineal laceration refers to a tissue tear involving the perineal region between the vaginal opening and the anus, including the skin, mucosa, muscles, and deeper soft tissues. It most commonly results from vaginal delivery, trauma, or other external forces. Based on the anatomical extent of injury, perineal lacerations are classified into four degrees:

Involves superficial tearing of the perineal body and/or vaginal mucosa;
Extends to the vaginal mucosa, perineal skin, and deeper submucosal tissues;
Involves the anal sphincter complex and is further subdivided according to the extent of external anal sphincter (EAS) involvement:
  Partial tear, involving less than 50% of the EAS;
  Complete tear, involving 50% or more of the EAS;
  Tear involving both the EAS and internal anal sphincter (IAS);
The most severe type, characterized by a complete third-degree tear with extension into the rectal mucosa (1).

Because vaginal delivery is the most common cause of perineal lacerations (2), third-degree and fourth-degree lacerations are also referred to as obstetric anal sphincter injuries (OASIS) (3). These injuries are not only commonly associated with severe vaginal delivery-related trauma, but also include operative vaginal delivery, fetal macrosomia, prolonged second stage of labor, and other factors causing excessive perineal tissue stretching (1). Although obstetric clinicians perform repair of these injuries, a substantial proportion of patients may still develop complications such as rectovaginal fistula, poor healing of the sphincter complex, or wound dehiscence (4), which can ultimately lead to chronic or neglected perineal lacerations. Chronic and neglected cases remain clinically challenging, particularly when diagnosis and repair are delayed for many years.

Chronic perineal lacerations may result in perineal body deformity or even loss, defects of the rectovaginal septum, and subsequent anal sphincter dysfunction. Some studies suggest that “women with clinically recognized anal sphincter injury are more than twice as likely to experience fecal incontinence compared with those without sphincter injury” (5). The risk is even higher in patients with grade IIIb to IV injuries (6), and fecal incontinence demonstrates a dose-dependent relationship, meaning that the more severe the tear, the higher the incidence of incontinence (7).

After menopause, reduced rectal propulsive function can worsen involuntary leakage of residual stool, significantly reducing quality of life. As a result, patients with chronic perineal lacerations often present primarily with fecal incontinence (8) in colorectal clinics. The key to improving fecal incontinence lies in anal sphincter repair and reconstructive surgery (5, 7, 9). For women whose initial lacerations were not adequately repaired, colorectal surgery often represents the “final stop” in the management of perineal laceration.

## 3 Analysis of perineal laceration repair techniques

Over the past several decades, surgical techniques for obstetric anal sphincter injury repair have become relatively standardized, with limited modifications in the fundamental principles of reconstruction. The two major approaches for external anal sphincter repair remain end-to-end approximation and overlapping sphincteroplasty (10).

The end-to-end repair technique (suturing the torn ends directly together without overlap) is more suitable for repair of external anal sphincter (EAS) injuries of grade IIIa and IIIb, as well as internal anal sphincter (IAS) injuries. Its advantages include precise anatomical alignment of muscle ends, low local postoperative tension, and good vascular supply; however, it has relatively weak resistance to tension.

The overlapping sphincteroplasty (overlapping the torn ends before suturing) is generally used for repair of IIIb external anal sphincter injuries (3). In addition, for fourth-degree perineal lacerations, repair requires careful reconstruction of the three layers of the anal canal—rectal mucosa, IAS, and EAS. Accordingly, different suturing techniques are applied at different levels: the rectal mucosa is first repaired with continuous or interrupted sutures, followed by end-to-end mattress suturing of the IAS, and finally overlapping repair of the EAS (3).

Some studies suggest that there is no significant difference in long-term outcomes between these two methods (7). However, anal sphincter injuries often present with asymmetric, irregular, and unequal muscle ends, and in chronic cases, the sphincter edges may become fibrotic and worn. In such situations, with the end-to-end technique, suture lines may align with muscle fiber orientation, increasing the risk of suture pull-through and surgical failure (11). Therefore, the overlapping sphincteroplasty is currently more widely recommended (7) and has been included in the American Society of Colon and Rectal Surgeons (ASCRS) textbook (12).

Because the overlapping sphincteroplasty produces a more robust sphincter complex and offers greater resistance to suture tearing through muscle fibers, it is widely used in clinical practice. However, this method also has several limitations:

1. Patients undergoing sphincter reconstructive repair are predominantly postmenopausal women, a phenomenon that may be associated with higher prevalence of fecal incontinence in this population (8). In many cases, perineal trauma occurred more than 20 years prior, leading to disuse atrophy of the anal sphincter muscles. Atrophic muscles have poorer blood supply and significantly reduced tensile strength compared with normal tissue. The overlapping sphincteroplasty introduces relatively high local tension, thereby significantly increasing the risk of suture dehiscence postoperatively.
2. In postmenopausal women, reduced estrogen levels lead to vaginal atrophy (13, 14), resulting in thinning of the rectovaginal septum. This thinning greatly increases the difficulty of dissecting between the vaginal and rectal walls. It also raises the risk of full-thickness injury, while impaired microcirculation of the mobilized mucosal flaps increases the likelihood of wound exposure, local necrosis, and infection, thereby contributing to surgical failure.
3. The overlapping sphincteroplasty creates a bulky “bulb-like” thickening at the muscle junction. This makes it more difficult to completely embed the sphincter complex within the rectovaginal septum. This issue is particularly pronounced in postmenopausal women with already thin rectovaginal tissue. Forced coverage of the muscle ends may result in local ischemia, excessive soft tissue tension, and fluid accumulation within the surgical field, all of which can contribute to failure of the repair.
4. Because the EAS has inherent resting tone (3), the overlapping sphincteroplasty creates a muscle junction that undergoes marked changes in local tension within a previously low-tension soft tissue environment. In addition, the bulbous repaired muscle junction is difficult to anchor securely at the apex of the dissected “pocket” within the rectovaginal space. As a result, the relatively mobile repair site is subjected to continuous mechanical stress during sphincter contraction, increasing tension at the suture line. This stress may be detrimental to wound healing: when patients voluntarily contract the pelvic floor, the anastomotic site is not effectively pulled in a cephalad direction, meaning that the more actively rehabilitation is performed, the greater the risk of wound dehiscence.
5. The overlapping sphincteroplasty requires layered reconstruction of the IAS, EAS, and rectal wall. Repair of the rectal wall may restrict local anal canal distensibility, producing a condition similar to anal stenosis. Moreover, the more secure the repair is attempted, the stricter the requirement for suture margins, which may increase the risk of wound disruption during postoperative defecation, leading to secondary infection and a higher risk of surgical failure.

## 4 Modified cross-suturing technique: the cross-lap scissors-leg method

Based on the limitations described above, a modified repair technique was proposed to reduce local stress concentration and unfavorable tension distribution at the muscle repair site (15, 16), as well as minimize the bulk of the reconstructed sphincter ends. This method is referred to as Cross-lap. It features cross-folding and suturing the two separated ends of the external anal sphincter in a scissor-leg crossing configuration.

**Figure 1:**
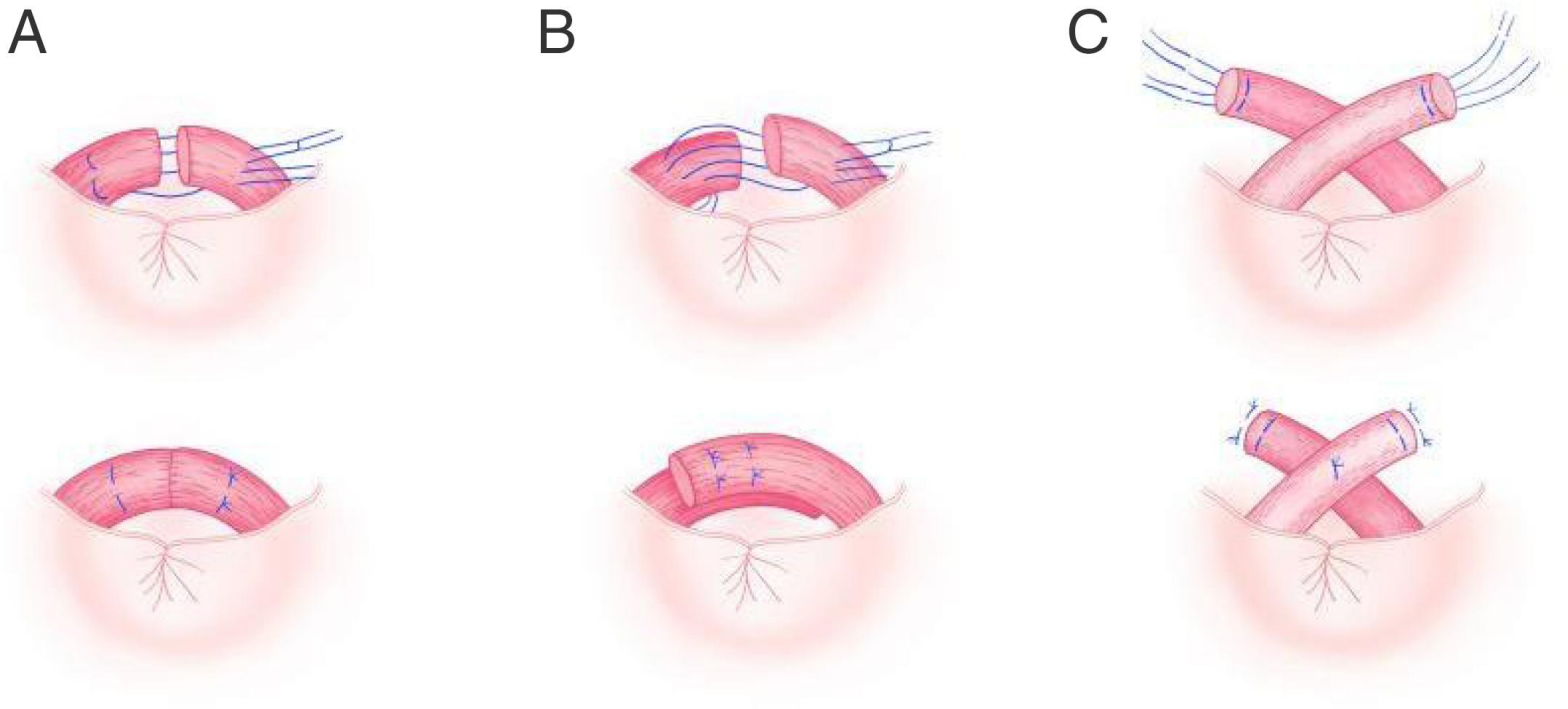
Schematic comparison of three anal sphincter repair techniques: end-to-end repair, overlapping sphincteroplasty and cross-lap suturing technique. **(A)** End-to-End Repair technique: suturing the torn ends directly together without overlap. **(B)** Overlapping sphincteroplasty: overlapping the torn ends before suturing. **(C)** Cross-lap Suturing Technique: cross-folding the torn edges in a “scissor-leg” manner.

To clarify the anatomical principles and technical distinctions of these three procedures, schematic diagrams depicting end-to-end repair, overlapping sphincteroplasty, and the Cross-lap suturing technique are presented in Figure 1.

The Cross-lap suturing technique was designed for patients with chronic or neglected obstetric anal sphincter injuries, particularly those with grade IIIb and grade IV perineal lacerations. In chronic cases, previous inadequate repair or delayed diagnosis may result in sphincter defect, fibrosis, muscle atrophy, and progressive fecal incontinence. The surgical procedure is described in detail below.

### 4.1 Preoperative preparation

Prior to repair, the degree of laceration must be accurately assessed.

Prophylactic administration of second-generation cephalosporins is recommended. Digital rectal examination should be performed before and after repair to assess postoperative anal sphincter tone (3).

Instrument preparation includes: sponges, vaginal gauze packing, Allis forceps(two), a needle holder, sharp toothed tissue forceps,3-0 synthetic absorbable sutures,2-0 synthetic absorbable sutures,and monofilament PDS sutures(multifilament sutures may increase infection risk (11).

### 4.2 Surgical technique

In a well-lit operating room, the patient is placed in the lithotomy position with full exposure of the laceration. The wound is irrigated with 1% povidone-iodine solution combined with 0.5% metronidazole solution, followed by sterile draping (11). Combined spinal-epidural anesthesia or spinal anesthesia is used. A diluted 1:1,000 epinephrine solution is injected into the rectovaginal septal tissues using a fine needle to reduce bleeding.

**Figure 2:**
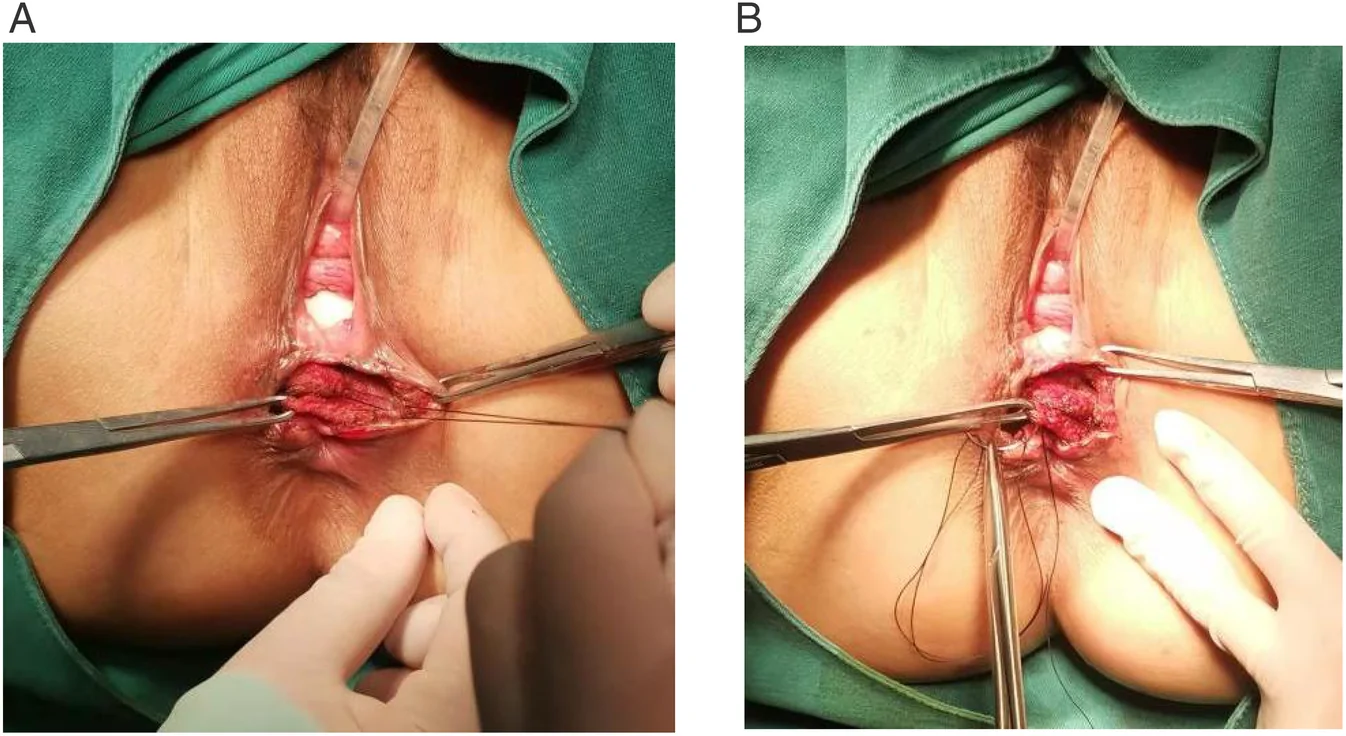
Schematic showing two key technical details of the cross-lap suturing technique. **(A)** The right sphincter end was drawn to the contralateral anchoring site, the combined complex of the opposite EAS and superficial transverse perineal muscle, and secured with a tension-reducing suture technique. **(B)** After fixation of the right sphincter end, the left sphincter stump is being anchored and sutured to the corresponding contralateral muscle bundle.

The repair follows strict anatomical layering and functional reconstruction principles, as outlined below:

1. Exposure and mobilization of sphincter ends:An incision is made along the rectovaginal mucosal junction, extending laterally on both sides toward the projected ends of the sphincter. The external anal sphincter ends are carefully identified and dissected free. Each end is grasped with Allis forceps and mobilized toward its contralateral target position (the combined complex of the opposite EAS and superficial transverse perineal muscle). Dissection continues until each muscle end can comfortably cross the midline. Blunt dissection is then used to separate approximately 2 cm of the rectovaginal septum to accommodate the repaired sphincter complex (Figure 2A).
2. Anal sphincter reconstruction (core step): Restoration of the anal sphincter is the key step for functional recovery. The two muscle ends are crossed and anchored to their contralateral target positions. The internal anal sphincter is first repaired using 3-0 PDS sutures in an end-to-end mattress fashion (3–4 stitches). The external anal sphincter is then repaired using at least four interrupted sutures with 2-0 absorbable material, taking care to avoid excessive traction that may cause ischemia or increased tension (Figure 2B).
3. Repair of the anterior rectal wall: Using a fine round needle and 3-0 absorbable sutures, interrupted inverting sutures are placed to repair the rectal wall defect. Starting from the midpoint of the incision, the rectal wall is approximated layer by layer to reconstruct a new rectal wall. A rectovaginal septal needle entry and mucosal exit technique is used. The reconstructed rectal wall includes submucosal and muscular layers to better resist tension. Suture spacing is approximately 0.5 cm to ensure adequate vascular supply. Repair proceeds from cephalad to caudad until approximately 1 cm beyond the anal verge. If anal stenosis is present, a posterior longitudinal incision or longitudinal incision with transverse closure technique may be used to relieve constriction.
4. Repair of the posterior vaginal wall: After exposure of the posterior vaginal wall, interrupted sutures using synthetic absorbable material are placed from cephalad to caudad, continuing until the level of the reconstructed posterior vaginal fornix is restored.
5. Perineal body reconstruction: The degree of perineal body defect is assessed, and the perineal body is reconstructed in one to two layers. Either a “coronal suture” technique or complete layered closure is performed to restore both anatomical appearance and structural support.

### 4.3 Postoperative care

A Foley catheter is maintained for 1 day. A high-calorie liquid diet is given for 1 day, followed by a residue-free semi-liquid diet for 3 days, then a regular diet.

For patients with Bristol stool types III–IV, no special intervention is required. For patients who preoperatively tend toward type I–II stools, lactulose 15 mL twice daily is administered for 7–10 days postoperatively. For type V–VII stools, smectite powder 3 g twice daily is used until stool consistency normalizes.

Wound assessment and appropriate management are performed at postoperative day 7 according to wound-care principles until complete healing is achieved. Clinical follow-up lasts for 3 months, with one or two visits each month. Pelvic-floor rehabilitation is considered safe to commence at three months postoperatively.

### 4.4 Surgical considerations

1. Maintain a clean operative field, ensure hemostasis, and avoid dead space formation.
2. Accurate tissue approximation is essential for successful repair. Postoperatively, maintain local hygiene and disinfection, and ensure stool is soft but not watery, with smooth passage.
3. Rectal wall suturing should be tight and evenly spaced. An inverting technique is preferred–the inner mucosal-submucosal layer is closely approximated to the defect edge, while the outer seromuscular layer is slightly offset to reinforce and reduce the risk of rectovaginal fistula.
4. Pelvic floor rehabilitation should only begin 3 months postoperatively after clinical evaluation. Training should start with gentle adaptive movements, with intensity increased slowly according to individual recovery status. Vigorous pelvic floor workouts are prohibited within the first three months after surgery.
5. In postmenopausal women with vaginal atrophy, preoperative administration of topical vaginal estrogen therapy (usually for 2 weeks, although shorter 7-day regimens have also been described) may enhance the elasticity and quality of the vaginal and rectovaginal septal tissues, facilitate precise surgical dissection, minimize tissue fragility and tearing, optimize wound healing, and potentially improve the success rate of repair.

### 4.5 Advantages of the cross-lap suturing technique

The primary advantage of the Cross-lap suturing technique is that, while still providing overlapping reinforcement of the sphincter ends, it reduces the total area and bulk of overlap. This method emphasizes anchoring each muscle end to the superior-lateral aspect of a “pocket” created in the dissected rectovaginal septum.

Two essential conditions must be met:

1. Fixation must cross the midline to the contralateral side;
2. The anchoring point corresponds anatomically to the deep surface of the contralateral labial erectile tissue, in the region between the superficial and deep transverse perineal muscles.

After Cross-lap repair, sphincter contraction still generates continence, but the resulting force vector is directed cephalad, thereby avoiding increased tension on the skin incision.

Several technical refinements may facilitate the procedure:

After rectovaginal septal dissection, a preliminary rectal wall suture can be placed on both sides of the midline using an inside-out technique (needle entering from outside the rectal wall and exiting on the opposite side). A 0.2 cm margin stitch can serve as a landmark for subsequent repair, simplifying the procedure.

For sphincter ends, fixation must cross the midline to the contralateral side to achieve the “cross” configuration. Crossing the midline improves biomechanical alignment and reduces dependence on perfect muscle edge symmetry. Each side is independently anchored to maximize functional preservation. This approach retains the concept of “folding repair”, while redirecting contractile forces cephalad rather than toward the incision line, thereby facilitating postoperative pelvic floor rehabilitation.

## 5 Illustrative case

The patient is a 58-year-old female, postmenopausal for 7 years, who presented to our colorectal surgery department with a 10-year history of recurrent anal prolapse during defecation. On admission, the patient reported symptoms of urgency, fecal seepage with loose stool, perianal moisture, anal fullness, and occasional abdominal pain, without anal pain. On physical examination: an arcuate defect was observed from the 12 o'clock position of the anal verge extending to the perineal region, with scar hyperplasia at the margins and no local erythema or tenderness. The condition was classified as a chronic grade IV perineal laceration. After obtaining informed consent, the patient underwent one week of local vaginal estrogen cream therapy, followed by the Cross-lap suturing technique.

Postoperative outcomes were assessed across multiple dimensions, including anorectal function, pain and discomfort, perineal appearance, and patient-reported quality of life. In this preliminary case, functional recovery was evaluated through clinical assessment of bowel control, bowel habits, fecal leakage, urgency, perianal discomfort, limitations in daily activities, and overall functional recovery. At the 8-week follow-up, the patient reported improved bowel control and relief of symptoms including anal fullness, perianal moisture, and discomfort. Although standardized scoring systems were not applied in this initial report, future studies should incorporate validated tools such as the Wexner score and quality-of-life measures for objective outcome evaluation.

Based on this case, we believe that the Cross-lap suturing technique has advantages in reducing tension at the muscle ends, lowering surgical difficulty, and facilitating postoperative recovery. However, its long-term clinical efficacy still requires further observation and additional clinical studies.

## Data Availability

The original contributions presented in the study are included in the article/Supplementary Material, further inquiries can be directed to the corresponding authors.
